# Risk factors for COVID-19 infections among health care workers in Ghana

**DOI:** 10.1371/journal.pone.0288242

**Published:** 2023-07-06

**Authors:** Margaret Lartey, Ernest Kenu, Vincent Jessey Ganu, Franklin Asiedu-Bekoe, Baafour Kofi Opoku, Alfred Yawson, Sally-Ann Ohene

**Affiliations:** 1 Department of Medicine and Therapeutics, University of Ghana Medical School, College of Health Sciences, University of Ghana, Accra, Ghana; 2 Department of Medicine, Korle Bu Teaching Hospital, Accra, Ghana; 3 Department of Epidemiology and Disease Control, School of Public Health, University of Ghana, Accra, Ghana; 4 Public Health Directorate, Ghana Health Service, Accra, Ghana; 5 Komfo Anokye Teaching Hospital, Kumasi, Ghana; 6 Department of Community Health, University of Ghana Medical School, College of Health Sciences, University of Ghana, Accra, Ghana; 7 World Health Organization, Accra, Ghana; University for Development Studies, GHANA

## Abstract

**Introduction:**

Health care workers (HCWs) are crucial to the fight against COVID-19 and are at risk of being infected. We sought to determine the risk factors and associations of COVID-19 among HCWs in Ghana during the period of the pandemic.

**Materials and methods:**

A case-control study was conducted using the WHO COVID-19 HCWs exposure risk assessment tool. A HCW was categorized as *“high risk”* for COVID-19 if s/he did not respond “always, as recommended” to adherence to Infection Prevention and Control (IPC) measures during a healthcare interaction. A HCW was categorized as *“low risk”* if s/he responded “always, as recommended” to adherence to IPC measures. We used univariate and multiple logistic regression models to determine associated risk factors. Statistical significance was set at 5%.

**Results:**

A total of 2402 HCWs were recruited and the mean age was 33.2±7.1 years. Almost 87% (1525/1745) of HCWs had high risk for COVID-19 infection. Risk factors identified were profession (doctor- aOR: 2.13, 95%CI: 1.54–2.94; radiographer—aOR: 1.16, 95% CI: 0.44–3.09)), presence of comorbidity (aOR: 1.89, 95%CI: 1.29–2.78), community exposure to virus (aOR: 1.26, 95% CI: 1.03–1.55), not performing hand hygiene before and after aseptic procedures performed (aOR: 1.6, 95% CI: 1.05–2.45); not frequently decontaminating high-touch surfaces always as recommended (aOR: 2.31, 95%CI: 1.65–3.22; p = 0.001) and contact with a confirmed COVID-19 patient (aOR: 1.39, 95% CI: 1.15–1.67). Among those who came into any form of contact with confirmed COVID-19 patient, providing direct care (aOR: 2.0, 95%CI: 1.36–2.94), face-to-face contact (aOR: 2.23, 95%CI: 1.41–3.51), contact with environment/materials used by COVID-19 patient (aOR: 2.25, 95%CI: 1.45–3.49) and presence during conduct of aerosol generating procedures (aOR: 2.73, 95%CI: 1.74–4.28) were associated with COVID-19 infection.

**Conclusion:**

Non-adherence to IPC guidelines puts HCWs at increased risk of COVID-19 infection thus ensuring IPC adherence is key to reducing this risk.

## Introduction

The Corona virus SARS-Cov-2, disease (COVID-19) continues to be a public health emergency since January 2020 [1]. Africa recorded its first case in February 2020 [2] with Nigeria reporting the first COVID-19 case in Sub-Saharan Africa [3]. Globally, as at the 7th June 2023, there have been 767,364,883 confirmed cases of COVID-19 with Africa recording 1.2% (9,532,788) of these cases [4]. The total reported deaths worldwide at the same time was 6,938,353 with Africa recording 2.5% (175,371) of these deaths. Ghana reported its first COVID-19 case on the 12th March 2020 and as at the 7th June 2023, it had 171,653 cases of COVID-19 with 1462 deaths [4].

The African region experienced the third wave of the COVID-19 infection over the period of June to August 2021 with seven countries then were still undergoing a resurgence [5]. Health care worker (HCW) infections continue to be reported and this is a concern as it imposes undue stress on the capacity of health system to respond to the disease [6], thus their personal safety needs to be ensured [7]. There have been several reports of COVID-19 infections among HCWs involved in clinical care [8–11]. The African continent had recorded 116,457 COVID-19 infections among HCWs as at 8^th^ August 2021 with South Africa accounting for 48% of these infections [5] arising from the first and second waves. But this increased to a total of 150,387 COVID-19 infections among HCWs in Africa as at 16^th^ January, 2022 with Tanzania (11%), Algeria (5.3%), Liberia (5.0%), Chad (5.0%) and Niger (4.2%) having the highest proportion of HCW infections by country at the time [12]. Ghana is one of the countries with high numbers of HCW COVID-19 infections and had reported 4763 HCW infections as at 16^th^ January, 2022 [12]. Only Eritrea had not reported any HCW infections. There have also been reports of deaths among HCWs from COVID-19 infection from China, Italy, Spain, USA and the United Kingdom [13–16].

The implementation of infection prevention and control (IPC) practices is very important regarding protection of HCWs from being infected [17, 18]. However, the lack or deficiency of personal protective equipment (PPE) in many clinical settings increases the risk of infections among these HCWs [11, 19]. Lack of implementation of IPC, inadequate personal protection of healthcare workers, pressure of treatment, work intensity and lack of rest have all been attributable factors to the risk of infection among HCWs [20]. No significant difference has been found with regards to the attack rate of COVID-19 infection among clinical and non-clinical HCWs [10].

There is little data on the epidemiology of COVID-19 infections among HCWs in Ghana.

A recently published study by Opoku et al. reported PPE use of 59.5% among HCWs in one region in Ghana [21]. Years of practice experience, type of health facility and adherence to IPC when in contact with patients were risk factors identified in the study [21]. A nationally representative study exploring epidemiological characteristics of COVID-19 infection among HCWs is useful in providing additional relevant information for policy and decision making. This study was therefore undertaken among HCWs in three regions in Ghana representing high, medium and low COVID-19 burden zones in Ghana to examine the epidemiological characteristics, risk factors and associated factors of COVID-19 infection.

## Materials and methods

### Study design and sites

A 1:1 case-control study was conducted in three regions (Ashanti, Western and Northern) representing high, medium and low burden zones in Ghana from 1^st^ November 2020 to 31^st^ January 2021.

### Study population

The study population was all HCWs actively providing healthcare services in the three selected regions who had received a test for COVID-19 with evidence of test results from any of the accredited COVID-19 test laboratories in Ghana and reported to the National database. For this study, health care workers (HCWs) were defined as all persons engaged in actions in the health facility with a primary intent to enhance health as defined by the WHO [22].

### Definitions

**Case.** Any health care worker with laboratory confirmed COVID-19 infection who worked in any health facility in Ghana from 12th March 2020 to 31st August 2020 was defined as a case.

#### Control

Any health worker with negative laboratory COVID-19 results who worked in any health facility in Ghana from 12th March 2020 to 31st August 2020 was defined as a control.

### Sample size and sampling procedure

A multi-stage sampling approach was used. The country was stratified into three zone High, Medium and low burden zones. This was based on the number of HCW cases recorded per region (high burden—≥600 HCW cases; medium burden– 300–599; low burden -≤299). For each zone, a simple random sampling was conducted to select one region per zone resulting in the selection of Ashanti region (High burden zone), Western region (Medium burden zone) and Northern region (Low burden zone).

A list of all HCWs tested for COVID-19 at all testing centers for the three regions and reported to the national database was obtained and reviewed. A sample of records for 1000 HCW cases was used based on the WHO recommendation [23]. The same number of controls was selected. A proportionate to size ratio of 0.6:0.3:0.1 for the high, medium and low burden regions respectively was used (See Table 1). In each selected region, we used generated random numbers to select the required sample size from the database of COVID-19 positive health care workers for that region. For each selected case, a HCW control matched by facility was selected from the same database who had tested negative for COVID-19.

**Table 1:** Distribution of sample sizes per area of burden for healthcare workers in Ghana, 2020.

Burden	Percentage	Sample size (cases)	Sample size (controls)
High	60	600	600
Medium	30	300	300
Low	10	100	100
**Total**	100	1000	1000

Each selected health worker case or control was contacted via phone by the research team and informed of the study. Arrangements for in-person meeting were made with those who agreed to participate in the study. Those declining were replaced with the next selected participant. Each participant provided a signed written informed consent before participating in the study.

### Data collection and tools

A structured data extraction form and the WHO COVID-19 health care workers exposure risk assessment tool [24] were used for this study.

Data obtained using the extraction form included patients’ socio-demographic variables (such as age, sex, marital status, level of education and profession), place of work, location of work, role at workplace, role in service provision, treatment received and clinical outcomes (discharged with or without disability).

The WHO COVID-19 health care workers exposure risk assessment tool [24] assesses community (2 items) and occupational/health system related exposure (5 items) to the COVID-19 virus; adherence to IPC during healthcare interactions (7 items); adherence to IPC when performing aerosol generating procedures (AGP) (6 items); accident with biological materials (1 item) and also risk categorization of HCWs.

The data was collected through questionnaire administration and all national COVID-19 protocols were strictly adhered to during the data collection period.

#### Community exposure

A HCW was considered to have community exposure to the COVID-19 virus if he or she responded “yes” to either having had any history of staying in the same household or classroom environment with a confirmed COVID-19 patient or had a history of traveling together in close proximity (within 1 meter) with a confirmed COVID-19 patient in any kind of conveyance.

#### Occupational/health system related exposure

A HCW was considered to have occupational/health system related exposure to the COVID-19 virus if he or she responded “yes” to having performed any of the following activities in the facility: direct care to a confirmed COVID-19 patient or the environment where the patient was cared for; face-to-face contact (within 1 meter) with a confirmed COVID-19; present when any procedure was performed on a patient, and involvement with healthcare interaction (paid or unpaid) in another facility.

#### Risk categorization

HCWs were categorized as *“high risk”* or *“low risk”* for COVID-19 virus infection. A HCW was categorized as *“high risk”* if he or she did not respond “always, as recommended” to adherence to IPC measures/protocols during a healthcare interaction with regards to the use, removal, and replacement of Personal Protective Equipment (PPE), performing hand hygiene and decontaminating high touch surfaces at least three times daily and also having any episode of accident with biological fluid/respiratory secretions. A HCW was categorized as *“low risk”* if he or she responded “always, as recommended” to adherence to IPC measures/protocols during a healthcare interaction with regards to the use, removal, and replacement of Personal Protective Equipment (PPE), performing hand hygiene and decontaminating high touch surfaces at least three times daily.

### Data analysis

Data was entered and cleaned in Microsoft Excel before it was exported into STATA version 15.0 (StataCorp LP, College Station, Texas, USA) for analysis. Descriptive analysis of categorical variables was presented in the form of frequencies and percentages as well as graphically using bar charts, pie charts and doughnut pie charts. The summary of age of HCWs was done with means and standard deviation.

Associations between host related, community exposure, health system/occupational- related risks and the outcome variables (COVID-19 infection and clinical outcome) was carried out using unadjusted and adjusted multiple logistic regression models. The crude odds ratio was computed for the identified host, health system and environment-related risk factors. The adjusted odds ratio for the different categories of risk factors were determined using the logistic regression model. Statistical significance was set at 5%.

### Ethical approval

Ethical approval was obtained from the Ghana Health Service Ethics Review Committee (ID GHS-ERC 008/06/20) and the Komfo Anokye Teaching Hospital Institutional Review Board (KATH-IRB/AP/142/20).

## Results

### Socio-demographic and clinical characteristics of HCWs

A total of 2402 HCWs from the three regions (Ashanti– 59.0%, Western– 32.7%, Northern– 8.3%) in Ghana participated in the study. The ratio of persons with COVID-19 infection (1201 cases) to non-COVID-19 infected health workers (1201 controls).

Majority (59.0%, 1418/2402) of the HCWs were females and the mean age of all HCWs was 33.2 ±7.1 years. More than half (58.3%, 1401/2402) of the participants were married/co-habiting and 92% (2215/2402) of the HCWs had some form of tertiary education. Nurses formed the majority (61.6%, 1484/2402) of study participants. Comorbid conditions occurred in 6% (149/2402) of HCWs. The proportion of cases who had comorbid conditions was double that of the controls (See Table 2).

**Table 2:** Socio-demographic and clinical characteristics of health care workers in Ghana, 2020.

	Case	Control	Total
	n(%)	n(%)	n(%)
**Region**			
High burden	716(59.6)	701(58.4)	1417(59.0)
Low burden	100(8.3)	100(8.3)	200(8.3)
Medium burden	385(32.1)	400(33.3)	785(32.7)
**Sex**			
Male	489(40.7)	495(41.2)	984(41.0)
Female	712(59.3)	706(58.8)	1418(59.0)
**Age**			
(Mean ± SD)	33.5 ± 7.5	32.8 ± 6.6	33.2 ± 7.1
20–29	398 (33.1)	421 (35.1)	819 (34.1)
30–39	610 (50.8)	625 (52)	1235 (51.4)
40–49	130 (10.8)	114 (9.5)	244 (10.2)
50–60	63 (5.3)	41 (3.4)	104 (4.3)
**Marital Status**			
Never Married	461(38.4)	504(42)	965(40.2)
Married/Co-habiting	719(59.9)	682(56.8)	1401(58.3)
Ever Married	21(1.8)	15(1.3)	36(1.5)
**Religion**			
Christianity	1098(91.4)	1097(91.3)	2195(91.4)
Islam	101(8.4)	100(8.3)	201(8.4)
Traditionalist	0 (0)	4(0.3)	4 (0.2)
Other	2 (0.2)	0 (0)	2 (0.1)
**Educational Level**			
Up to Primary	28(2.3)	38(3.2)	66(2.8)
Secondary	55(4.6)	66(5.5)	121(5)
Tertiary	1118(93.1)	1097(91.3)	2215(92.2)
**Profession**			
Nurse	733 (61.0)	753 (62.7)	1484 (61.9)
Doctor	140 (11.7)	66 (5.5)	206 (8.6)
Paramedic/transport	21 (1.8)	25 (2.1)	46 (1.9)
Pharmacist	21 (1.8)	27 (2.3)	48 (2.0)
Lab scientist/technic	48 (4.0)	67 (5.6)	115 (4.8)
Radiographer	9 (0.7)	8 (0.7)	17 (0.7)
Cleaner/Orderly	35 (2.9)	69 (5.8)	104 (4.3)
Others(specify)	68 (5.7)	61 (5.1)	129 (5.4)
Admin/ account staff	104 (8.7)	109 (9.1)	212 (8.9)
Disease control officer	22 (1.8)	16 (1.3)	38 (1.6)
**Comorbidity**			
No	1103(91.8)	1150(95.8)	2253(93.8)
Yes	98(8.2)	51(4.3)	149(6.2)
**Number of comorbidities** ^**¶**^			
One	88(89.8)	47(92.2)	135(90.6)
Two*	10(10.2)	4(7.8)	14(9.4)
**Hypertension** ^**¶**^			
No	50(51)	30(58.8)	80(53.7)
Yes	48(49)	21(41.2)	69(46.3)
**Diabetes**			
No	81(82.7)	42(82.4)	123(82.6)
Yes	17(17.4)	9(17.7)	26(17.5)
**Kidney disease** ^**¶**^			
No	97(99)	50(98)	147(98.7)
Yes	1(1)	1(2)	2(1.3)
**HIV** ^**¶**^			
No	96(98)	51(100)	147(98.7)
Yes	2(2)	0(0)	2(1.3)
**Other Comorbidities** ^**¶**^			
No	58(59.2)	27(52.9)	85(57.1)
Yes	40(40.8)	24(47.1)	64(42.9)

n: Frequency, %: Column Percentage, SD: Standard Deviation, uOR: Unadjusted Odds Ratio, Other Comorbidities conditions included ulcer, asthma, sickle cell, Rheumatoid Arthritis

**¶**: Applicable to only those with comorbid conditions

*: combinations included: Hypertension & Diabetes (n = 9), Hypertension & HIV (n = 1), Hypertension & Kidney disease (n = 1) hypertension & ulcer (n = 1), hypertension & sickle cell (n = 1)

### Community exposure to COVID-19 virus infection among HCWs

Approximately 26% (631/2402) of all HCWs had community exposure to COVID-19 Virus (Table 2).

Thirty percent (359/1201) of cases and 22.2% (266/1201) of controls had community exposure to COVID-19 virus infection (Table 3). Twenty six percent (625/2402) of HCWs lived in the same house with a confirmed COVID-19 person and 0.3% (7/2402) travelled together in close proximity with a confirmed COVID-19 patient in any kind of conveyance to the health facility (Table 3).

**Table 3:** Community exposure to COVID-19 Virus among health care workers in Ghana, 2020.

	Case	Control	Total	Binary logistic regression		
	n(%)	n(%)	n(%)	UOR	95% CI	P-value
**Staying in same house COVID-19 person**						
No	842(70.1)	935(77.9)	1777(74.0)	1.00		
Yes	359(29.9)	266(22.2)	625(26.0)	1.50	1.25–1.8	<0.001
**Relationship with positive COVID-19 patient** ^**#**^						
Parent/Sibling	5 (1.4)	6 (2.3)	11 (1.8)	1.00		
Spouse/Children	12 (3.4)	8 (3)	20 (3.2)	1.80	0.41–7.95	0.438
Auntie/Uncle	3 (0.8)	1 (0.4)	4 (0.6)	3.60	0.28–46.36	0.326
Patient	241 (67.3)	130 (48.7)	371 (59.4)	2.22	0.67–7.42	0.194
Colleague worker	69 (19.3)	99 (37.1)	168 (26.9)	0.84	0.25–2.85	0.775
other^¥^	28 (7.8)	23 (8.7)	51 (8.1)	1.46	0.39–5.41	0.570
**HCW have history of traveling together in close proximity with a confirmed COVID-19 patient in any kind of conveyance**						
No	1200(99.9)	1195(99.5)	2395(99.7)	1.00		
Yes	1(0.1)	6(0.5)	7(0.3)	0.17	0.02–1.38	0.097

other^**¥**^ includes boyfriend, driver, housemate/neighbor among others

**#**: Applicable to only persons who stayed in the same house with a COVID-19 person.

### Occupational-related exposure to COVID-19 virus infection among health care workers

Exposure to COVID-19 virus infection in health facilities occurred in 19% (451/2402) of health care workers.

Fifty-eight percent (1389/2402) of all health care workers indicated they had come into contact (within 1 meter) with a confirmed COVID-19 patient in the health facility. For those who came into contact with a confirmed COVID-19 patient, the risk factors identified were provision of direct care to the patient (aOR: 2.0, 95%CI: 1.36–2.9; p = <0.001); face-to-face contact with the patient (aOR: 2.23, 95%CI: 1.41–3.51; p = <0.001); performing aerosol generating procedures (AGP) for the patient (aOR: 2.73, 95%CI: 1.74–4.28; p = <0.001) and having contact with materials/environment used by the patient (aOR: 2.73, 95%CI: 1.74–4.28; p = <0.001). Provision of locum services was not an identified risk factor and was not statistically significant (aOR: 0.53, 95%CI: 0.26–1.07; p = <0.077)

### Risk categorization for COVID-19 virus infection among health care workers in Ghana

Complete data on adherence to IPC measures/protocols during a healthcare interaction with any patient was available for 1745 health care workers. The proportion of health workers who were at high risk of COVID-19 infection in providing care to patients was 87.4% (1525/1745).

### Adherence to infection prevention and control and PPE use during patient interactions

On assessment of Infection Prevention and Control (IPC) activities, not performing hand hygiene before and after any clean or aseptic procedure (aOR: 1.6, 95%CI: 1.05–2.45; p = 0.03) and not frequently decontaminating high-touch surfaces (aOR:2.31, 95%CI: 1.65–3.22; p = 0.001) always as recommended were significant predictors of having COVID-19 infection.

During HCW interaction with patients, the use of PPEs as recommended in guidelines among HCWs were medical mask (47.6% (831/1745) face shield/goggles/protective glasses 20.5% (358/1745) and disposable gown 29.4% (513/1745) (Table 4). The proportion of controls that always used face shield/goggles/protective glasses as recommended was 7.3% more than the cases. Only 41.8% (343/820) of controls compared to 35.7% (330/925) of cases always replaced PPE as recommended but associations were not statistically significant (Table 4).

**Table 4:** PPE use and adherence to infection prevention and control measures among HCWs during health care interactions with patients in Ghana, 2020.

	Control	Case	Total	Adjusted Logistic Regression	
	N = 820 n (%)	N = 925 n (%)	N = 1745 n (%)	aOR (95% CI)	P-value
**Medical mask use**					
Always as recommended	401 (48.90)	430 (46.49)	831 (47.62)	1	
Not always as recommended	419 (51.10)	495 (53.51)	914 (52.38)	1.0(0.72–1.41)	0.986
**Face shield or goggles/protective glasses use**					
Always as recommended	200 (24.39)	158 (17.08)	358 (20.52)	1	
Not always as recommended	620 (75.61)	767 (82.92)	1387 (79.48)	1.31(0.92–1.88)	0.138
**Disposable gown use**					
Always as recommended	272 (33.17)	241 (26.05)	513 (29.40)	1	
Not always as recommended	548 (66.83)	684 (73.95)	1232 (70.60)	1.07(0.74–1.55)	0.728
**Replaced your PPE according to protocol use**					
Always as recommended	343 (41.83)	330 (35.68)	673 (38.57)	1	
Not always as recommended	477 (58.17)	595 (64.32)	1072 (61.43)	1.21(0.91–1.6)	0.188
**Perform hand hygiene before and after any clean or aseptic procedure was performed**					
Always as recommended	383 (46.71)	382 (41.30)	765 (43.84)	1	
Not always as recommended	437 (53.29)	543 (58.70)	980 (56.16)	1.60(1.05–2.45)	0.030
**Perform hand hygiene after exposure to body fluid**					
Always as recommended	386 (47.07)	404 (43.68)	790 (45.27)	1	
Not always as recommended	434 (52.93)	521 (56.32)	955 (54.73)	0.94(0.63–1.39)	0.752
**Perform hand hygiene after touching patient’s surroundings**					
Always as recommended	279 (34.02)	260 (28.11)	539 (30.89)	1	
Not always as recommended	541 (65.98)	665 (71.89)	1206 (69.11)	0.84(0.58–1.23)	0.371
**High-touch surfaces decontaminated frequently**					
Always as recommended	233 (28.41)	156 (16.86)	389 (22.29)	1	
Not always as recommended	587 (71.59)	769 (83.14)	1356 (77.71)	2.31(1.65–3.22)	0.001

n: Frequency, %: Column Percentage, SD: Standard Deviation, aOR: adjusted Odds Ratio

The proportion of controls that always performed hand hygiene before and after any clean or aseptic procedure as was 5.4% more than the cases. The odds of being infected with COVID-19 was 1.6 times higher among HCWs who did not always perform hand hygiene before and after any clean or aseptic procedure compared to those who always did (95% CI: 1.05–2.45, p = 0.023). The odds of being infected with COVID-19 was 2.3 times higher among HCWs who did not always frequently decontaminate high-touch surfaces compared to those who always did (95% CI: 1.56–2.46, p = <0.001) (Table 4).

### COVID -19 symptoms and duration

**Fig 1:**
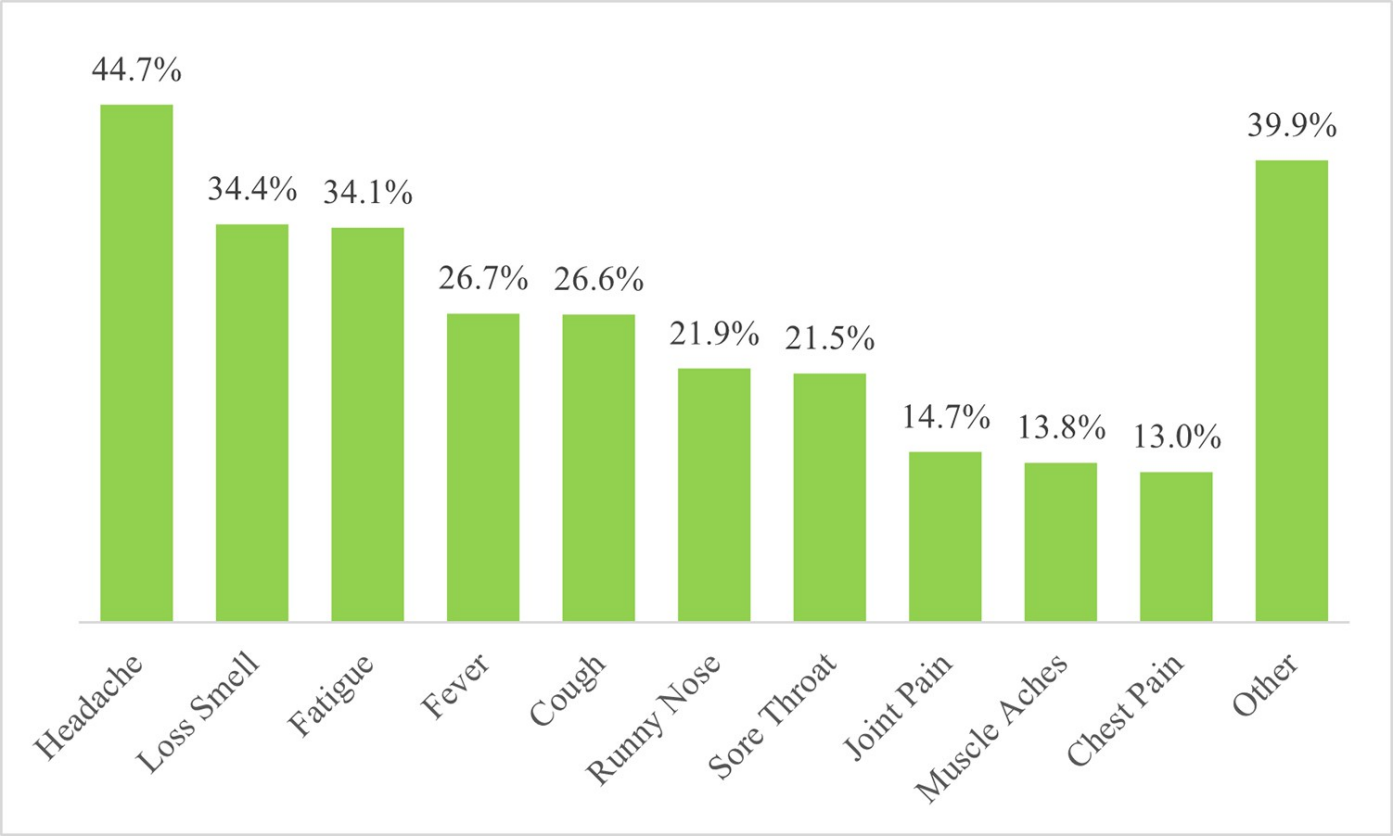
Top ten symptoms among health care workers infected with COVID-19 in Ghana.

In assessing the symptoms exhibited by cases, headache was the predominant (44.7%, 537/1201) reported symptom and lasted for a median of 5 days (Table 5). About one-third of the cases lost their sense of smell (34.4%, 413/1201) with a similar proportion experiencing fatigue (34.1%, 410/1201). The two symptoms each lasted for a median duration of 5 days (Table 5). Fever was experienced by a quarter (26.7%, 321/1201) of the health workers with COVID-19 infection during the period under study (Fig 1).

**Table 5:** COVID -19 symptoms and duration among infected health care workers in Ghana, 2020.

	Case	Duration in days
	n(%)	Median (LQ, UQ)
Headache	537(44.7)	5(3, 7)
Loss Smell	413(34.4)	5(4, 10)
Fatigue	410(34.1)	5(4, 7)
Fever	321(26.7)	5(3, 7)
Cough	320(26.6)	5(3, 7)
Runny Nose	263(21.9)	5(3, 7)
Sore Throat	258(21.5)	5(3, 7)
Joint Pain	177(14.7)	5(3, 7)
Muscle Aches	166(13.8)	5(3, 7)
Chest Pain	156(13)	5(3, 7.5)
Short Breath	123(10.2)	4(3, 8)
Diarrhoea	99(8.2)	4(2, 5)
Nausea	82(6.8)	3(2, 5)
Abd Pain	43(3.6)	4(3, 7)
Ear Pain	29(2.4)	3(3, 7)
Wheezing	29(2.4)	7(4, 7)
Dark Urine	27(2.3)	4(3, 7)
Confusion	23(1.9)	7(3, 14)
Dark Stool	12(1)	3(3, 6)
Haemoptysis	5(0.4)	2(1, 2)
Seizures	5(0.4)	2(2, 2)
Aggressiveness	4(0.3)	7(3, 19)

n: Frequency, %: Column Percentage, LQ: Lower quartile, UQ: Upper quartile

**Fig 2:**
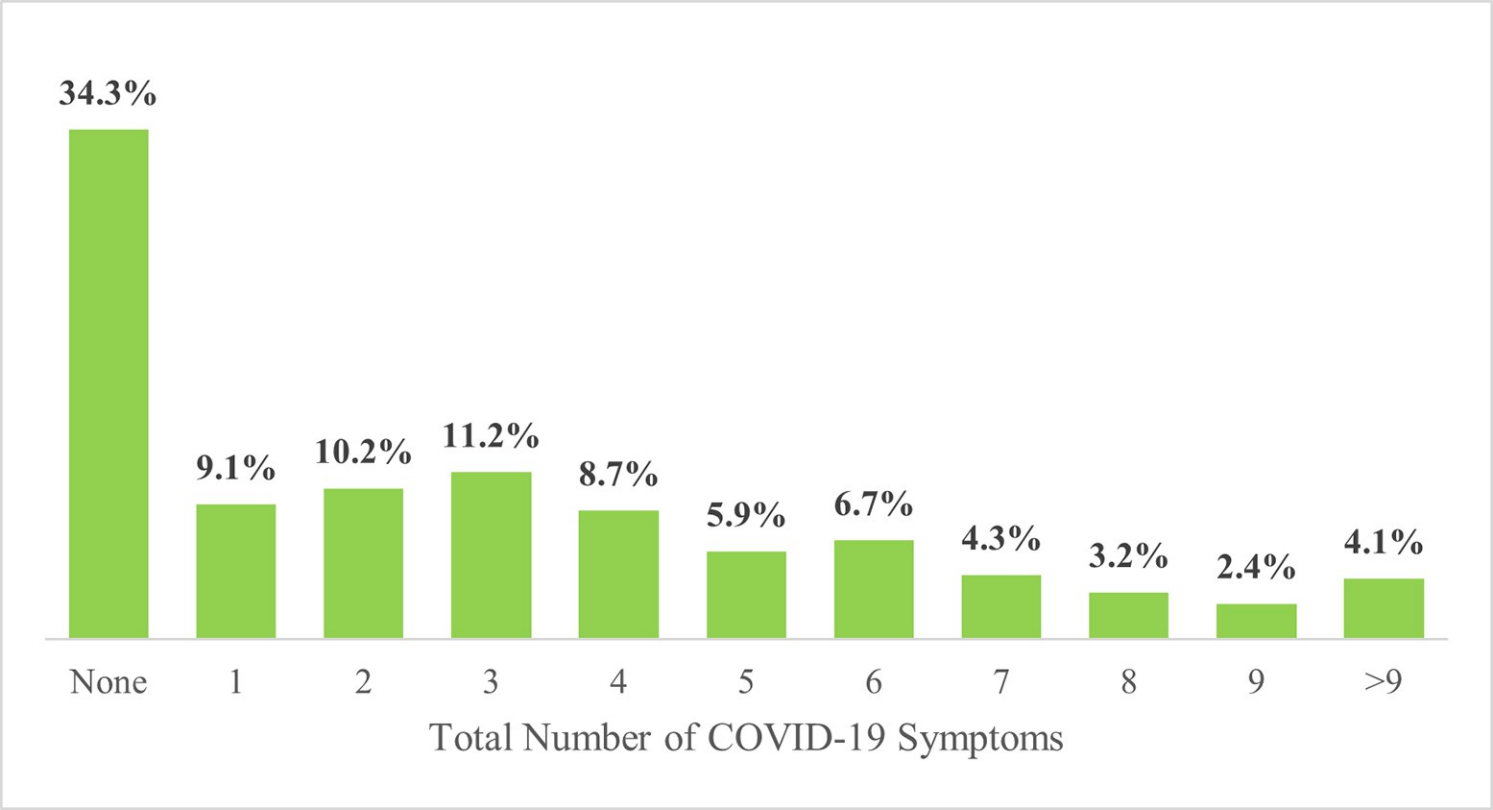
Number of COVID-19 symptoms experienced by health care workers infected with COVID-19 in Ghana.

About a third (34.3%, 412/1201) of the health care workers with COVID-19 infection were asymptomatic while another third (39.2%, 471) of them showed at most four symptoms (Fig 2).

### Clinical care received and outcome

More than three-quarters (89.7%, n = 1077) of the health workers self-isolated at home after contracting COVID-19 whilst 9.3% (112/1201) had in-patient quarantine. Only 1% (12/1201) of infected HCWs required admission. Out of those who required admission, 0.4% (5 /12) of them were admitted to the intensive care unit (ICU). About nine out of every ten (93%, n = 1121) selected health worker who contracted COVID-19 was discharged without any immediate complications.

### Association between host-related characteristics, community exposure, health facility exposure risks and COVID-19 infection among health care workers in selected regions of Ghana

Profession of respondents, previous contact with a confirmed COVID-19 patient, staying in the same house with COVID-19 patient and having co-morbid conditions were significantly associated with COVID status of respondents. The odds of contracting COVID-19 infection were 2.1 times higher among medical doctors compared to nurses (aOR: 2.12, 95%CI:1.54–2.93) (Table 6). The Cleaner/Orderly had 59% reduced odds of contracting COVID-19 infection compared to nurses (aOR: 0.41, 95%CI: 0.23–0.73). Getting into contact with a confirmed COVID-19 patient was associated with 39% increased odds of getting infected with COVID-19. The odds of contracting COVID-19 infection was 90% more among health workers with comorbid conditions than those without such conditions (aOR: 1.90, 95%CI: 1.29–2.78). With regards to community exposure, health workers who were exposed had 0.28 higher odds of contracting COVID -19 compared to those who were not exposed (aOR: 1.28, 95%CI: 1.03–1.59) (Table 6).

**Table 6:** Association between COVID-19 infection and host-related characteristics, community exposure, health facility exposure risks among health workers in Ghana, 2020.

		Binary Logistic regression	
	n	aOR(95% CI)	P-value
**Profession**			
Nurse	1484	1.00	
Doctor	206	2.12 (1.54–2.93)	0.001
Paramedic/transport	46	0.79 (0.42–1.47)	0.454
Pharmacist	48	0.86 (0.48–1.56)	0.627
Lab scientist/technician	115	0.78 (0.52–1.16)	0.221
Radiographer	17	1.16 (0.44–3.08)	0.768
Cleaner/Orderly	104	0.41 (0.23–0.73)	0.003
Others(specify)	129	1.06 (0.72–1.58)	0.761
Admin/ account staff	212	1.04 (0.77–1.4)	0.808
Disease control officer	38	1.41 (0.72–2.79)	0.317
**Contact with a confirmed COVID-19 patient**			
No	1013	1.00	
Yes	1389	1.39 (1.15–1.67)	0.001
**Comorbidity**			
No	1777	1.00	
Yes	625	1.90 (1.29–2.78)	0.001
**Community exposure**			
Unexposed	1771	1.00	
Exposed	631	1.28 (1.03–1.59)	0.027
**Health related risk**			
Low risk	102	1.00	
High risk	2300	1.08 (0.7–1.68)	0.716

n: Frequency, aOR: Adjusted Odds Ratio, CI: Confidence Interval

### Association between clinical outcome of COVID-19 infection and host-related characteristics, community exposure, health facility exposure risks and among health workers in selected regions of Ghana

Having a comorbidity was the only significant predictor of clinical outcome of COVID-19 infection.

The odds of being discharged with complication among health works with comorbidity was 2.5 times higher than those without any comorbidity (aOR: 2.48, 95%CI: 1.16–5.30).

## Discussion

This study provides some insight into the epidemiology of COVID-19 infection among HCWs during the first two phases of the pandemic in Ghana. An increased risk of infection was associated with non-adherence to IPC protocols. The study revealed that 26.3% and 18.8% of HCWs had community and health system/occupational-related exposure to the COVID-19 virus respectively. Almost 87% of HCWs had high risk for COVID-19 infection due to non-adherence to strict IPC measures. Only 29.4% and 47.6% of HCWs used face shields/goggles and medical mask respectively as recommended always during patient interactions in work environment. For HCWs with COVID-19 infection, majority (61%) were nurses. Headache and loss of smell were the predominant symptoms among COVID-19 infected HCWs. Only 1% of HCWs with COVID-19 infection required admission to the hospital with 40% of those admitted requiring ICU admissions.

The HCWs in this study were generally a young population with a mean age of 33.2 years with 59% being female. This is expected as nurses made up the largest health care force in the country and majority were female.

Nurses formed the majority (61%) of HCWs infected with COVID-19 from our study. This finding was consistent with results from a systematic review of databases undertaken till May 2020 where COVID-19 infections were mainly in nurses (38.6%) [6]. Similarly, nurses formed the majority of COVID infected cadre of HCWs in Wuhan (52.1%) and Iran (51.3%) [25, 26]. In the set-up of our healthcare system, the nurses are usually the commonest cadre of HCWs to interact with patients in out-patient engagement, in-patient care, drug administration and performing procedures which increases their exposure risk to infection [27–29]. These reasons likely contributed to the differential effects of occupation types in infection status as shown in all the studies with the nurses being most affected.

Compared to nurses, doctors and cleaners had significant higher odds of getting COVID-19 infection. This may be due to the nature of their work encountering patients in closed spaces or proximity which increases their risk of acquiring the infection. In relation to disease control officers, they may have been exposed during cleaning and dusting activities on exposed surfaces.

The prevalence of co-morbidities among the HCWs in our study was 6.2% with hypertension being the commonest co-morbidity. This was low compared to reports of co-morbid prevalence of 18.4% from a systematic and meta-analysis of seven studies by Gholami et al. [30] and 38% reported among HCWs in United States [31] although hypertension was also the com reported co-morbidity by Ghalomi et al. Our study participants were mostly young as indicated by their mean age which could be the reason for the lower rate of comorbidities among HCWs.

Our study revealed low occupational exposure to COVID-19 virus with a rate of 18.8%. This is in contrast to other studies in Ghana and the United States where the level of occupational exposure was reported to be 80.4% and 55% respectively [31, 32]. Compared to the other study in Ghana, the high occupational exposure reported was as a result of the study being conducted in a designated COVID-19 treatment center where all patients were infected and therefore the exposure was high. The differences in the low rate of occupational exposure compared to that of US is likely due to the low prevalence of the COVID-19 infection in Ghana and Africa in general compared to the US. Although occupational exposure risk was low among our participants, COVID-19 infection risk still exists as the pandemic persists with newer, more virulent and easily transmissible variants.

Our study also revealed that 87.4% of all HCWs were at risk of COVID-19 infection as they did not comply with PPE usage as always recommended during health care interactions with patients in the health facilities. This was similar to findings by Opoku et al. [21] where PPE use was among only 59.5% of HCWs. However, our study finding was is in contrast to the previous study by Ashinyo et al. [32] in Ghana where only 14% of HCWs were at high risk of COVID-19 infection. The difference in risk may be due to availability of PPEs as well as increased compliance in the treatment center as HCWs in those centers were aware they were dealing with infected patients. This brings to the fore the point that HCWs need to apply standard appropriate PPEs to every encounter and upscale as the condition demands. In the era of a pandemic, to treat every patient with the same precautions they will use when treating COVID-19 infected patients. The study did not identify PPE shortages as a risk and this could be due to the fact that the pandemic has gone through a second wave and most countries had largely addressed the issue of inadequate supplies.

Compliance to IPC measures during the pandemic was low among our participants with less than 50% compliance with hand hygiene practices during patient interactions, before or after performing procedures, and using PPEs as always recommended. Afeng-Nkansah et al. and Alah et al. reported similar findings where only 44.1% and 50% respectively of HCWs were fully compliant with IPC measures (PPE usage and hand hygiene). Another study by Ashinyo et al. [33] reported 97.5% for hand hygiene and PPE usage among HCWs in Ghana. The contrasting findings by Ashinyo et al. can be attributed to the setting of their study which were designated COVID-19 treatment centers where availability of IPC materials, training of staff on IPC measures as well as monitoring of IPC adherence will be ensured. In addition, HCWs in the treatment centers will make conscious efforts to adhere to IPC measures knowing the risk as they provide care to confirmed COVID-19 patients. For this risk to be mitigated, compliance of HCWs to IPC measures as well as availability of PPEs needs to be ensured by hospital authorities.

This demonstrates that HCWs in non-COVID treatment health facilities will need support in terms of refresher trainings on IPC together with behavior change interventions to mitigate the risk of COVID-19 infection.

Almost 34% of HCWs in our study were asymptomatic. Similarly, 35.5% of infected HCWs were reported to be asymptomatic in a study by Sabetian et al. in 2020 [25]. The predominant symptoms among symptomatic HCWs in our study were headache (44.7%) and loss of smell (34.4%), both lasting a median of 5 days. Previous studies in Iran [25] reported myalgia (46%) and cough (45.5%) as the predominant symptoms whilst reports from systematic review from Gholami et al. reported fever (27.5%) and cough (26.1%) as the predominant symptoms [30]. The numbers of asymptomatic patients endorse the time held public health responses of physical distancing and masking to break the transmission cycle. Also, symptoms vary but for our setting, headache and loss of smell were considered key in our suspicion of COVID-19 infection when interacting with patients. However, anosmia is currently not a major symptom of the Omicron variant due to the changing trend in symptomatology since the beginning of the pandemic [34]. It is crucial for public health surveillance team to frequently review data for change in trends and continuously engage and update decision makers to ensure timely information sharing and action taking in our response to the pandemic.

Only 12 (1%) HCWs in our study were admitted to the hospital out of which 5 (41%) were admitted into the ICU. This admission rate was lower than a previous study from Iran (5.5%) with no ICU admissions and US (10%) with some ICU admissions [25, 31]. Gholami et al. also reported a hospitalization rate of 15.1% in systematic review of 13 studies [30]. Similar high hospitalization and ICU admission rates in China in the early stages of the pandemic in 2020 [35, 36]. Majority (93%) of our patients who were admitted were discharged home without any complications. The low hospitalization rate among our study participants may be attributed to the younger population of our study participants, the lower numbers of existing co-morbidities among our participants and the low prevalence in sub-Saharan Africa in general. In the early stage of the pandemic, the aged and those with co-morbidities formed a significant part of hospitalization. Thus, majority of our participants did not experience severe forms of the disease.

Beyond COVID-19, strengthening the health system to ensure robust IPC practices in health care facilities will serve overall to benefit not only HCWs but also patients and visitors through reduction in health care associated infections [37].

Though this study is representative as it spanned the 3 major regions in the country, there were some limitations. The case-control approach of the study may have resulted in recall bias that may affect responses received from participants thereby urging caution in interpreting findings. Another limitation is that the study did not evaluate the support available to HCWs for risk mitigation such as trainings and safety of HCWs.

## Conclusion

There was high occupational exposure to COVID-19 virus among HCWs in health facilities. Doctors and cleaners were at more risk of exposure compared to nurses. Activities such as aerosol generating procedures, provision of direct care, face-to-face contact as well as contact with environment or materials used by patients with COVID-19 were key risks for exposure. Non-adherence to IPC guidelines puts HCWs at increased risk of COVID-19 infection.

To minimize the risk of COVID-19 infection and also protect the HCW, health institutions need to ensure robust IPC practices in facilities, ensure consistent supply of needed PPEs and implementation of hand hygiene measures. Effective monitoring and supervision of IPC implementation measures should be instituted to minimize risk of COVID-19 transmission to ensure a healthy and effective workforce.

## Supporting information

S1 Data (XLS)
